# Building health-promoting school environments: a longitudinal qualitative study of the Explo’Santé program in France

**DOI:** 10.3389/fpubh.2026.1857951

**Published:** 2026-05-26

**Authors:** Florence Carrouel, Adeline Darlington-Bernard, Corélie Salque, Guylène Tchuendem, Emma André, Matteo Olivo, Emily Darlington

**Affiliations:** Université Lyon 1, P2S UR4129, Lyon, France

**Keywords:** education, health-supportive environments, implementation, life skills, school health promotion

## Abstract

**Introduction:**

Health inequalities among children and adolescents remain a major public health concern, with schools identified as key settings for integrated, context-sensitive interventions. The Explo’Santé program was developed in France to promote life skills and foster health-supportive school environments by targeting school-level determinants, including relational dynamics, organizational processes, and classroom environments.

**Methods:**

A longitudinal qualitative study was conducted between 2022 and 2025 across six French districts, based on 36 semi-structured interviews with primary and middle school teachers involved in program implementation. Data were analyzed using thematic analysis to explore perceived environmental changes and contextual mechanisms influencing implementation.

**Results:**

Six main themes emerged, including spatial conditions, peer climate, teacher–student relationships, organizational flexibility, pedagogical formats, and institutional embedding. In primary schools, stable classroom environments enabled gradual symbolic and relational transformations, with improved emotional expression, peer interactions, and integration into routine practices. In contrast, middle schools were characterized by organizational fragmentation, reduced temporal and relational continuity, and more heterogeneous student engagement, limiting program integration. Sustainability appeared dependent on contextual alignment, coordination, and institutional support.

**Conclusion:**

The findings highlight that school-based health promotion effects are context-dependent and shaped by interactions between develop-mental stage and institutional structure. Sustaining psychosocial gains appears to depend on maintaining relational and organizational continuity across educational stages, as well as on stronger integration of health promotion into formal school structures.

## 1 Introduction

Health inequalities among children and adolescents remain a major public health concern worldwide. A substantial proportion of young people are affected by preventable or modifiable health conditions from early life, including mental disorders, overweight, and behavioral risk factors that tend to persist into adulthood. Indeed, nearly 15% of young people aged 10–19 experience a mental health disorder, accounting for 13% of the global burden of disease in this age group (1). In parallel, overweight and obesity rates among children and adolescents continue to rise globally, with pronounced social and geographic disparities that reflect broader structural inequalities (2). Together, these findings illustrate the early accumulation of health inequalities and underscore the need for early, integrated and context-sensitive public health interventions targeting children and adolescents (3).

Schools are widely recognized as a strategic setting for such interventions (4). As environments attended by nearly all children during critical developmental periods, schools shape not only academic trajectories but also socialization, well-being and health-related behaviors. From a public health perspective, schools offer a unique opportunity to reach diverse populations and to address social gradients in health (5–7). In response, the World Health Organization has promoted the Health Promoting Schools (HPS) framework, which advocates a whole-school approach integrating health into school environments, pedagogical practices, participation and governance, rather than limiting action to individual health education (8). Within this perspective, the development of healthy school environments, understood as school settings where the physical, social and organizational conditions actively support health and well-being (9), is considered a key mechanism to promote well-being and reduce social inequalities.

However, translating whole-school frameworks into measurable and sustainable change remains challenging due to the complexity of multi-level interactions, strong contextual dependency and variability in implementation across schools (10, 11). Methodological syntheses emphasize that school-based health promotion programs constitute complex public health interventions, the effects of which emerge from interactions between program components, institutional structures, professional practices and local contexts (8, 12, 13). As a result, the effects observed are often modest, heterogeneous and highly dependent on implementation conditions (14). This complexity partly explains why quantitative evaluations frequently yield mixed or context-specific findings, particularly when outcomes extend beyond individual behaviors to include environmental, organizational or relational dimensions (15, 16). In this perspective, longitudinal qualitative approaches offer a complementary and particularly relevant lens, as they enable the exploration of evolving processes, underlying mechanisms, and contextual dynamics that shape how complex interventions are implemented and experienced over time. Such an approach is particularly relevant to capture how implementation processes and contextual mechanisms evolve across educational stages, especially during key transitions such as the shift from primary to middle school. Within this context, the development of psychosocial or “life skills” (LS) has become a prominent focus of school-based health promotion (17). LS are commonly mobilized as resources enabling young people to cope with everyday challenges, interact with their social environment and engage with health-related determinants (18). However, in a whole-school perspective, LS are increasingly viewed not merely as individual coping mechanisms, but as relational assets capable of fostering a positive school climate and driving environmental change. Recent work highlights substantial heterogeneity in how LS are defined, operationalized and implemented across school contexts, as well as the difficulty of linking individual skill development to broader transformations of school environments (19, 20). This underscores the importance of examining not only outcomes, but also the processes through which LS interventions are integrated into school life.

In France, LS education has been flagged as a national priority notably in schools (21). The Explo’Santé program was developed within this framework as a school-based health promotion intervention combining classroom sessions focused on LS development with activities encouraging collective reflection on well-being and school environments (22). This program consists of a structured series of classroom sessions delivered over a three-year period, from 4th to 6th grade, across six French districts. It includes 10 sessions per year and focuses on LS development through interactive and experiential activities (e.g., emotional expression, peer interaction, and decision-making). These sessions are complemented by collective activities encouraging reflection on school environments, such as exploratory walks and group discussions, aiming to foster both individual competencies and collective environmental change within the school setting. Rather than aiming solely to change individual behavior, Explo’Santé was conceived as an intervention seeking to act on multiple dimensions of the school setting, in line with whole-school health promotion principles (22). Quantitative evaluations of Explo’Santé have provided important baseline and short-term outcome data. A cross-sectional study described levels of LS and related variables such as self-efficacy, life satisfaction, and health literacy among fourth-grade students, revealing substantial variability across students and educational contexts (23, 24). A subsequent three-year longitudinal evaluation reported mixed and modest changes, with effects varying according to school context and implementation conditions rather than showing uniform or robust improvements (25). Such findings are consistent with the literature on complex public health interventions and underscore the importance of approaches that capture contextual, organizational, and relational processes (16).

In this perspective, a qualitative approach is particularly relevant to explore how Explo’Santé is implemented and experienced within school settings, and how it may contribute to changes that are not immediately quantifiable. The present study therefore seeks to deepen the understanding of the processes and contextual mechanisms through which Explo’Santé may influence school environments.

Specifically, the aim of this study is to examine how the Explo’Santé program contributes to the development of health-supportive school environments in France, from the perspective of the teachers involved in its implementation. Using a qualitative longitudinal design, this research explores the perceived changes across the physical, social, pedagogical, organizational and governance-related dimensions of the school environment, and analyses the contextual factors that facilitate or constrain these transformations.

## 2 Materials and methods

### 2.1 Study design

This study adopted a longitudinal qualitative design conducted between 2022 and 2025 in metropolitan France. Although the study followed a longitudinal structure across program phases, different teachers were interviewed at each time point. The design therefore corresponds to a repeated cross-sectional qualitative approach, allowing comparison across educational stages rather than tracking individual trajectories over time. The study was informed by theoretical frameworks including the HPS framework, socio-ecological models, and implementation science perspectives, which conceptualize school health interventions as multi-level processes shaped by interactions between individuals, organizations, and institutional systems. It was embedded within a broader school-based health promotion research program coordinated by the Parcours Santé Systémique (Health Systemic Process) (P2S, UR4129) research unit at Université Claude Bernard Lyon 1, in partnership with the Ligue contre le Cancer (French League against Cancer), which was responsible for program implementation in schools.

The study is reported in accordance with the Consolidated Criteria for Reporting Qualitative Research (COREQ) guidelines recommended by the EQUATOR Network (Enhancing the QUAlity and Transparency of Health Research) (26).

### 2.2 Research team and reflexivity

#### 2.2.1 Researchers’ characteristics and roles

Data collection and analysis were performed by trained qualitative researchers (ADB, CS, MO), with expertise in health promotion, school-based interventions, and qualitative methodologies. The investigators consisted of two women (ADB, CS) and one man (MO). They held master’s degrees and were doctoral students with a research interest in LS and health promotion but had no hierarchical or evaluative role vis-à-vis the participating teachers.

Researchers involved in data collection were not responsible for implementing the Explo’Santé program in schools, thereby limiting potential role conflict and power imbalance.

The researchers’ backgrounds and interest in life skills may have influenced both data collection and interpretation, particularly in the attention given to relational and organizational dimensions of the program. To address this, reflexive discussions were conducted throughout the analytical process to critically examine emerging interpretations and limit potential bias. This approach aimed to ensure that findings remained grounded in participants’ accounts rather than researchers’ preconceptions.

#### 2.2.2 Relationship with participants

No prior personal or professional relationship existed between interviewers and participants before the study. Participants were informed that the research aimed to explore their experiences and perceptions of the program, and that their responses would have no impact on their professional evaluation or on future program deployment.

### 2.3 Study setting and context

The study was conducted in six French districts selected to reflect a diversity of territorial, socio-economic and educational contexts: Ardèche, Corrèze, Hérault, Ille-et-Vilaine, Loire and Loire-Atlantique. These districts include rural, peri-urban and urban areas, as well as schools located in socially contrasted environments.

The Explo’Santé program was implemented in primary schools (4th and 5th grade) and middle schools (6th grade) within these districts.

### 2.4 Participants and recruitment

Participants were invited via institutional communication channels.

Participants were included if: (i) they were aged 18 years or older, (ii) they were teachers in 4th, 5th or 6th grade during program implementation; (iii) they were directly involved in hosting the Explo’Santé program; (iv) they provided oral informed consent and (v) they had authorized the recording and processing of their voice.

Participants were excluded if: (i) they were teachers not involved in the program; (ii) they were staff with non-teaching roles (e.g., principals, school nurses, counsellors); (iii) they were teachers from other grade levels or; (i) they were teachers absent for extended periods during implementation.

Basic demographic and professional characteristics of participants were collected, including gender, educational qualification and school context. These variables were selected to ensure diversity in institutional and professional positioning across educational levels and territories.

More detailed individual characteristics, such as age or years of teaching experience, were not systematically collected. This reflects the design of the study, which primarily aimed to explore contextual and organizational mechanisms of program implementation rather than to analyze variations linked to individual teacher profiles. In addition, given the relatively small number of participants per district and educational level, the collection of highly specific individual data could have increased the risk of indirect identification.

### 2.5 Sampling

The study used a purposive sampling strategy (27), based on voluntary participation, to ensure diversity in educational level (4th, 5th or 6th grade), school context and territorial setting. This strategy aimed to capture variability across key dimensions relevant to the research question, including educational stage, school organization, and local context, which are expected to shape the implementation and perception of the program. Participants were identified through institutional communication channels in collaboration with local coordinators involved in program deployment. While participation was voluntary, purposive selection ensured inclusion of teachers from diverse contexts and educational stages, enabling meaningful comparison across school levels.

### 2.6 Sample size

The sample size was defined *a priori* in the Explo’Santé research protocol to align with the program’s longitudinal design and territorial organization. For the qualitative component, individual interviews were conducted with two teachers per district at each data collection time.

Interviews were carried out at three time points corresponding to program implementation phases (end of the 4th grade, end of the 5th grade, and end of the 6th grade). This design resulted in a total of 36 individual teacher interviews across the study period. Each teacher was interviewed only once even in case of participation in the Explo’Santé program during 2 years.

This predefined sample size was intended to ensure comparability across territories, school levels and time points, while allowing sufficient flexibility to reflect the realities of school staffing and program implementation.

Although the number of interviews was predefined, data collection and analysis were iterative, and thematic saturation was reached, with no new themes emerging across the main analytical domains.

### 2.7 Development of the interview guide

The interview guide was developed based on the study objectives and informed by relevant theoretical frameworks, including the HPS framework, the Whole School Approach, and socio-ecological and implementation science perspectives (22). It covered key domains such as program implementation, contextual influences, perceived effects, and sustainability. The guide was used consistently across the three data collection waves, with minor adaptations to reflect the timing of program exposure. Additional details are provided in Supplementary material 1.

### 2.8 Data collection

Data were collected through individual semi-structured interviews conducted in France at three phases of program implementation with 4th grade teachers (November 2023–January 2024), 5th grade teachers (May–July 2024), and 6th grade teachers (April–June 2025). Interviews were conducted in a one-to-one format via videoconference (Cisco WebEx) by three trained qualitative researchers (ADB, CS, MO) (Table 1).

**Table 1:** Overview of data collection.

**Dimension**	**Description**
Participants	36 teachers
Educational levels	4th grade, 5th grade, 6th grade
Time points	T1: Nov 2023–Jan 2024 (4th grade) T2: May–Jul 2024 (5th grade) T3: Apr–Jun 2025 (6th grade)
Data collection method	Individual semi-structured interviews
Mode	Videoconference (Cisco WebEx)
Interviewers	Three trained qualitative researchers (ADB, CS, MO)
Data recorded	Audio-recorded and transcribed verbatim

A total of 36 teachers participated. Interviews were audio-recorded, transcribed verbatim, and anonymized. The same interview guide was used across time points, with minor adaptations reflecting program exposure. Additional details on the interview procedures are provided in Supplementary material 2.

### 2.9 Data analysis

Data analysis was conducted using a hypothetico-deductive approach and coding methods. Thematic analysis was conducted following the approach proposed by Braun and Clarke (28), which was selected as a flexible method suitable for identifying patterns across heterogeneous school contexts and capturing both anticipated and emergent dimensions of a complex intervention. This approach is particularly relevant for exploring organizational, relational, and contextual dynamics. The five-step methodology framework for thematic analysis, as proposed by Braun and Clarke, was applied (28).

1. Familiarization with the data: This step involved reading and re-reading the transcripts to fully immerse in and understand the content.
2. Generating initial codes: Raw data (verbatim transcripts) were transformed into initial meaningful codes. These were then organized into themes aligned with the research questions and hypotheses. Then, relevant excerpts were isolated to create semantically decontextualized, independent units for thematic grouping. This was followed by recontextualization to restore meaning across grouped codes and themes. Coding combined deductive and inductive approaches. An initial coding framework was informed by the study objectives and theoretical frameworks, including the HPS framework and implementation science perspectives, and was iteratively refined through engagement with the data. Coding was conducted independently by three researchers (GT, ADB and FC) and discussed to ensure consistency and resolve discrepancies.
3. Searching for themes: The data were reorganized through a hierarchical structure of concepts to generate broader themes. These themes were enriched and interconnected via constant comparison, ultimately forming a structured framework based on the initial research questions. During this stage, the coding framework was reflexively reviewed against the experiential elements recurrently emphasized by participants. This ensured that themes were not only derived from a deductive structure and meaning-making processes. Themes were developed through an iterative process of constant comparison across participants, educational levels, and time points, allowing the identification of both shared patterns and context-specific dynamics.
4. Reviewing of themes: Themes were reviewed and refined collaboratively. This collaborative process ensured the internal coherence of themes and alignment with the study objectives.
5. Defining and naming themes: Finally, finer coding was used to illustrate the themes with specific examples. At least one verbatim is selected for each theme.
6. Analytical rigor was supported through triangulation of researchers, iterative discussions, and comparison across contexts and time points.

### 2.10 Data validation

Several procedures were applied to support reliability. Interview transcripts were systematically reviewed for accuracy by a second researcher. Data analysis was conducted using a thematic approach by three researchers (GT, ADB and FC). Sample adequacy was defined *a priori* by the study protocol and subsequently assessed through thematic saturation, which was monitored during analysis. By the completion of the planned interviews, no substantially new themes emerged within the main analytical domains.

Analytical robustness was further supported through comparative analysis across educational levels, territorial contexts and data collection waves. Verbatim quotations are presented to illustrate key themes and to allow readers to assess the linkage between empirical material and interpretative claims.

Participants were not asked to review transcripts or validate the final thematic structure. However, their accounts were analyzed iteratively and remained central to the refinement of interpretations throughout the analytical process.

### 2.11 Ethical approval

The study complied with the Declaration of Helsinki, General Data Protection Regulation and French data protection regulations.

Ethical approval was obtained from the Terre d’Éthique Ethics Committee of the University Hospital of Saint-Étienne (France), reference IRBN962023/CHUSTE.

All participants provided written informed consent prior to participation. Participation was voluntary, and no financial or material incentives were offered. Data were collected and processed anonymously in compliance with applicable data protection standards.

## 3 Results

### 3.1 Participants

Participant characteristics and data collection are presented in Table 2. Aggregated demographic and professional characteristics (gender, educational qualification and school context) are reported to contextualize the sample, while detailed individual information is provided in Supplementary Table 1. For each grade level, two teachers per district were interviewed (*n* = 12 per grade). Overall, 36 teachers were interviewed across the three grade levels. The average duration of each interview was 39′33 (min 21′00–max 73′00).

**Table 2:** Participant characteristics.

**Characteristic**	**Description**
Total participants	36 teachers
Gender	31 women 5 men
Educational qualification	24 Recruitment Exam for Elementary School Teachers 12 Certificate of Qualification for Secondary School Teaching
School context	26 rural 12 urban
Districts	6 French districts
Participants per district	2 teachers per grade level
Data collection phases	T1:4th grade (*n* = 12) T2: 5th grade (*n* = 12) T3: 6th grade (*n* = 12)
Interview duration	Mean: 39 min Range: 21–73 min

### 3.2 Major themes and sub themes emerging

The qualitative analysis explored how the Explo’Santé program was perceived to influence school environments across different educational levels. It focused on identifying key dimensions of change and contextual mechanisms shaping implementation. In particular, the analysis examined how these changes relate to broader organizational and environmental transformations within school settings, including institutional structures, governance processes, and system-level constraints. This qualitative analysis revealed six main themes:

Theme 1. Spatial Configuration and Material Conditions.

Theme 2. Peer Climate and Horizontal Social Regulation.

Theme 3. Teacher–Student Relationship and Authority Reconfiguration.

Theme 4. Organizational Flexibility and Temporal Governance.

Theme 5. Pedagogical Formats and Age-Related Engagement.

Theme 6. Institutional Embedding, System Integration and Sustainability.

#### 3.2.1 Spatial configuration and material conditions: symbolic adjustments in primary school and structural constraints in middle school

In primary school settings, teachers do not report major structural redesign of school spaces. There were no timetable overhauls nor institutional spatial reforms. However, they describe the emergence of symbolic micro-transformations within otherwise stable classroom environments. These findings suggest that environmental change operates primarily through incremental and symbolic adjustments embedded in stable organizational contexts, rather than through formal structural transformation.

This transformation often began with the development of a critical awareness of the school environment itself:

“It enabled the students to learn to discover their environment […] The exploratory walk, because it was the students themselves who said what might not be working well in the school.” (5th grade teacher 5).

Students’ observations targeted concrete material elements:

“They clearly see what should be improved in the playground, or under the covered area, the posters that are half torn down, they say they should be removed, that it looks dirty […] the toilets are too small or smell bad, that sort of thing.” (5th grade teacher 2).

These observations indicate that the intervention fosters environmental appropriation, whereby students actively reinterpret their surroundings and identify actionable points of improvement.

In some cases, this reflection extended to adult behavior and lead to visible action:

“We had put up a small sign saying ‘Smoke-free space’ […] Now, we point it out to parents who used to smoke and who continue to smoke” (5th grade teacher 4).

Such actions suggest that symbolic changes can extend beyond the classroom and influence social norms within the broader school community, including interactions with adults.

Material supports also played a structuring role:

“There were the visual resources that… that helped a lot, that we could use… that we kept… that remained as resources, that stayed as materials in the classroom.” (4th grade teacher 11)

This highlights the importance of materializing abstract concepts within the physical environment, facilitating both understanding and spatial appropriation.

Some spatial limitations were nonetheless mentioned:

“We didn’t really have the space in the classroom to do it.” (4th grade teacher 9)

Despite these constraints, the primary classroom remained structurally stable, supporting gradual internalization of program principles through repeated and localized adaptations.

In middle school, by contrast, spatial and temporal fragmentation were central constraints. These findings indicate that organizational and spatial discontinuities limit the continuity of intervention processes and constrain the integration of program activities into everyday school functioning.

Sessions were compressed and discontinuous:

“an hour, bearing in mind it’s not really an hour, it’s 55 minutes. […] Well, in the end, there’s actually about 45 minutes left”. (6th grade teacher 5)

These temporal constraints reflect broader institutional scheduling structures, which limit flexibility and reduce the capacity to integrate complex interventions within existing organizational systems.

Students’ constant movement between rooms reinforced discontinuity:

“It’s true students change classroom every hour… […] They change buildings. […]there are several of us teachers involved (…)”. 6th grade teacher 8

These features reflect a fragmented organizational model, which contrasts with the continuity observed in primary school and reduces opportunities for sustained engagement.

Classroom configurations also influenced implementation:

“It quickly became chaotic in small classrooms where they don’t have any space.” (6th grade teacher 10)

“[…] in a flexible classroom […] it was more practical […] than in a traditional classroom”. (6th grade teacher 9)

This contrast suggests that spatial configuration plays a key role in shaping interaction patterns and the effectiveness of participatory pedagogical approaches.

Thus, while primary teachers described adaptive micro-transformations within stable environments, middle school teachers faced structural fragmentation that constrained spatial continuity. Overall, these findings point to a shift from symbolically mediated change in stable contexts to structurally constrained implementation in fragmented organizational settings, highlighting the importance of institutional configuration in shaping intervention outcomes.

#### 3.2.2 Peer climate and horizontal social regulation: stabilization in primary school and heterogeneous appropriation in middle school

In primary schools, teachers described observable improvements in students’ capacity to verbalize emotions and regulate interpersonal tensions among peers. These findings suggest that the intervention contributes to the development of horizontal regulatory processes, where peer interactions increasingly rely on verbalization and shared norms rather than immediate reactive behaviors.

A teacher illustrated the shift from factual to emotional framing:

“Before […] it was more down-to-earth, ‘he pushed me,’ and so on, and afterwards, […] they were more able to put words into what they were feeling. […] it was ‘well, he pushed me, so that’s how it made me feel,’” (4th grade teacher 10).

This shift from descriptive to emotional language reflects a transformation in how social interactions are processed, indicating a move toward more reflexive and mediated forms of peer regulation.

Teachers also described reusing program tools beyond sessions:

“We reused certain things that had been introduced during the sessions […] such as the ‘feelings weather report,’ and so on, which naturally stayed with the children.” (5th grade teacher 1)

These observations indicate a process of internalization, whereby tools initially introduced within structured sessions become embedded in everyday classroom interactions and sustained beyond the intervention context.

Conflict resolution evolved:

“It was a challenging class […] and they were more able to control themselves. […] I find that they communicated better with each other […] they didn’t go straight […] into conflict. And they were more able […] to talk things through, to say what they were feeling […] which before, they weren’t able to do at all.” (4th grade teacher 11)

These findings suggest a shift from adult-mediated conflict resolution toward more autonomous peer-based regulation, supported by shared communicative tools and norms.

Group cohesion was also perceived as strengthened, suggesting that changes in individual interactions may extend to broader collective dynamics within the classroom:

“We didn’t have small groups of children anymore, it was the entire class setting up games in the playground.” (4th grade teacher 5)

At the collective level:

“I find that the 5th graders are less unpleasant than those in previous years. […] We assumed this was linked to the program.” (5th grade teacher 8)

Taken together, these elements indicate that the intervention contributes to the emergence of a more cohesive and emotionally expressive peer climate, supported by shared relational resources.

In middle school, peer dynamics were perceived as more heterogeneous. These findings suggest that the effects of the intervention become more variable and context-dependent, reflecting increased social differentiation and complexity during early adolescence.

Teachers noted meaningful engagement with themes such as influence and harassment:

“On the topic of influence […] they grasped quite a lot of things […] in relation to social networks, in relation to popular people.” (6th grade teacher 7)

These examples indicate that engagement is enhanced when the intervention content aligns with socially salient issues, particularly those related to peer status, influence, and identity formation.

However, engagement varied:

“With those who are still a bit more childish, it works well, but with the others, there’s […] a bit of teasing. They feel like they’re being lectured and they don’t really see the point of it…” (6th grade teacher 4)

This variability suggests that peer climate in middle school is structured by differentiated developmental trajectories and social positioning, which can facilitate or hinder the appropriation of intervention content.

Overall, while primary school settings appear to support the stabilization of shared peer norms and relational practices, middle school contexts are characterized by more fragmented and heterogeneous dynamics, limiting the uniform diffusion of these processes across students.

#### 3.2.3 Teacher–student relationship and authority reconfiguration: emotional trust in primary school and boundary negotiation in middle school

In primary school, Explo’Santé opened a distinct emotional dialogue space between adults/teachers and students. These findings suggest that the intervention facilitates a reconfiguration of teacher–student relationships, shifting from a predominantly evaluative framework toward a more relational and emotionally responsive mode of interaction.

Teachers described moments of unexpected disclosure:

“[…] things really happened, I mean there were children who… who were able to open up like that […] it was really interesting […] sometimes it was surprising […]”. (4th grade teacher 6)

These accounts indicate that the presence of alternative interactional settings within the classroom enables forms of expression that are less accessible within routine pedagogical structures.

Teachers also reported a shift in posture:

“During the sessions, we were more in an observer role. And that’s so rare it’s actually nice […] I’m discovering them, really.” (5th grade teacher 2)

This shift reflects a partial decentering of the teacher’s traditional authority role, allowing the emergence of a more facilitative and supportive posture, which may enhance relational trust and student engagement.

At middle school level, teachers described a reduction of hierarchical distance. These findings suggest that similar relational dynamics are activated in secondary education, but within a more complex institutional and developmental context:

“It’s the first time, even though I’ve been a homeroom teacher for years, that they told me: “there’s 27 of us, and the advantage we have in this class is that it’s like at home… we can speak without being judged”.” (6th grade teacher 3)

These elements indicate that the intervention can temporarily reshape relational norms by reducing perceived hierarchical distance and fostering more open communication.

However, this relational openness generated ambivalence. Some students extended assertiveness into contestation:

“We also have students who challenge things […] since we taught them that they needed to express their emotions. […] Yes, but there, Miss, you didn’t do that.” (6th grade teacher 1)

These situations highlight a tension between empowerment and institutional order, where increased expressive capacity may challenge established authority structures.

Teachers emphasized the need to reassert boundaries:

“So, very often, we have to reset the boundaries and say that, […] when the teacher has said something, well, you have to accept it and respect what has been said.” (6th grade teacher 2)

This need for boundary reassertion suggests that relational transformations remain contingent on institutional constraints and developmental stage, particularly during early adolescence.

Overall, while primary school settings appear to support the emergence of emotionally grounded and trust-based relationships within a stable framework, middle school contexts reveal a more complex reconfiguration, where increased relational openness coexists with tensions around authority and norm regulation. These findings indicate that the impact of the intervention on teacher–student relationships is mediated by both organizational structures and developmental dynamics, highlighting the need for context-sensitive adaptation of relational practices.

#### 3.2.4 Organizational flexibility and temporal governance

Insights into how the program was implemented in practice across school contexts shed light on how it unfolded in everyday settings. The findings highlight how organizational conditions shape the implementation of the Explo’Santé program, revealing the influence of structural and temporal factors on its integration across school levels.

In primary school, the program was perceived as dense:

“It still involves ten sessions, um, I mean it’s something that’s a bit, um, that’s somewhat cumbersome to… to put in place.” (4th grade teacher 2)

These observations suggest that program intensity may represent both a constraint and a driver of engagement, requiring local adaptation to fit within existing pedagogical routines.

However, organizational flexibility allowed integration:

“I adjusted a few things, but that’s part of being a teacher.” (5th grade teacher 12).

These findings indicate that the primary school organizational model, characterized by teacher autonomy and flexible scheduling, enables local adjustments that facilitate program integration.

Integration with curriculum was noted:

“It also fits into our curriculum in science and civic and moral education.” (4th grade teacher 10).

This suggests that alignment with existing curricular structures acts as a key facilitator, allowing the program to be embedded within routine teaching practices rather than perceived as an external add-on.

At middle school level, rigidity dominated, with time constraints and coordination requirements limiting flexibility. These findings indicate that organizational structure plays a central role in shaping implementation capacity.

“an hour, bearing in mind it’s not really an hour, it’s actually 55 minutes and the students leave when the bell rings.” (6th grade teacher 1)

“In primary school, the teacher decides. In middle school, it’s more complicated. You need the whole team’s agreement.” (6th grade teacher 5)

These elements highlight a shift toward a more rigid and segmented organizational structure, where time is tightly regulated and distributed across multiple actors, limiting local adaptability.

Coordination became necessary:

“You need to have the approval of all the teachers.” (6th grade teacher 7)

This need for collective coordination suggests that implementation in middle school is less dependent on individual initiative and more constrained by institutional governance and interprofessional alignment.

Overall, these findings point to a contrast between flexible and teacher-driven organizational models in primary school and more regulated, collective, and fragmented governance structures in middle school. This shift appears to influence the capacity to integrate the intervention, suggesting that temporal governance and decision-making structures play a central role in shaping implementation processes and sustainability.

#### 3.2.5 Pedagogical formats and age-related engagement

Insights into students’ observed engagement across school levels highlight the central role of pedagogical formats in shaping their involvement in the program. The findings show how forms of participation, levels of interaction, and perceived relevance influence engagement differently in primary and middle school contexts.

In primary school, experiential activities were key factors of Explo’Santé implementation:

“It was really an exchange, and they were actively involved.” (5th grade teacher 5)

“[…] an activity they really liked a lot. It was when they had a paper plate taped to their back and had to ask their classmates to write, um, five qualities about them.” (4th grade teacher 5)

These findings suggest that engagement in primary school is strongly driven by participatory and emotionally salient pedagogical formats, which facilitate active involvement and reinforce positive peer interactions.

Materialization supported engagement:

“As soon as it was, well, visually and concretely materialized, um… I know the children really liked it a lot.” (4th grade teacher 12)

This indicates that translating abstract concepts into tangible and visual forms plays a key role in supporting both comprehension and engagement in younger students.

More passive sessions reduced attention:

“And sometimes there were sessions with a bit less… activity, and so on, where the students were a little more passive. And it’s true that we sometimes felt, and even the students felt that it was a bit long.” (4th grade teacher 10)

These observations highlight the importance of maintaining an active pedagogical structure, suggesting that engagement is closely linked to the level of interaction and participation embedded in the session design.

Teachers valued ready-to-use materials:

“She arrived with the materials, everything was organized, everything was photocopied […] it allowed us to save time.” (4th grade teacher 8)

This reflects the role of structured pedagogical support in facilitating implementation, particularly by reducing the organizational burden on teachers and enhancing feasibility in everyday practice.

At middle school level, engagement is more conditional and depends on students’ perception of relevance, particularly when sessions connect to concrete middle school experiences:

“On the topic of influence […] they grasped quite a lot of things when it comes to that whole influence dynamic and power play, in relation to social networks, in relation to popular people. […] Empathy, friendship, influence — that’s really, um… what’s at stake in middle school.” (6th grade teacher 1)

This suggests that engagement increases when content aligns with socially meaningful issues related to identity, peer influence, and everyday experiences.

But resistance emerged when activities were perceived as misaligned:

“They kind of felt… infantilized at the beginning […] they really didn’t want to expose their [personal] problems.” (6th grade teacher 3)

This resistance suggests that certain pedagogical formats may be perceived as misaligned with developmental expectations, leading to disengagement when activities are interpreted as inappropriate or insufficiently adapted to adolescent norms.

Overall, these results point to a shift from participation-driven engagement in primary school to relevance-driven engagement in middle school. This transition highlights the importance of adapting pedagogical formats to developmental stages, suggesting that the effectiveness of intervention activities depends not only on their design but also on their perceived legitimacy within the social and cognitive frameworks of students.

#### 3.2.6 Institutional embedding, system integration and sustainability

This theme examines how the program is integrated within institutional structures and how system-level factors influence its sustainability across educational contexts.

In primary school, one teacher emphasizes the progressive clarity of the proposed framework:

“This project was proposed to us, we were given… the overall framework, and then later on we received, um… the detailed session plans […] we understood gradually […] where we were heading.” (4th grade teacher 9)

These findings suggest that progressive structuring and clarity of program design support appropriation by teachers, facilitating integration into routine practices over time.

The quality of coordination and the structured nature of the system appear to be important facilitators:

“The fact that the sessions were already fully prepared and designed […] we didn’t have any preparation to do. […] It’s all ready to go, yes, but it’s well thought out.” (5th grade teacher 5)

These elements indicate that external structuring and logistical support reduce implementation burden and enhance feasibility, thereby acting as key facilitators of program adoption at the classroom level.

However, extension beyond school remained limited:

“The school can’t do this on its own […] we need to find a way to reach… into families as well and get them, um… involved in this. And… that’s what… that’s what was missing.” (4th grade teacher 11)

These observations highlight a limitation in the program’s reach, suggesting that while classroom-level integration may be achieved, broader systemic embedding remains constrained by weak links with external stakeholders and community contexts.

Teachers would like the sustainability of Explo’Santé:

“… I would like it to go further […] so that it can continue over time.” (5th grade teacher 3)

“We’re going to discuss it […] at the school council meeting with the city council. And I think things will be put in place.” (5th grade teacher 5)

These statements suggest that sustainability at the primary school level depends on continued support and extension beyond initial implementation, highlighting the importance of maintaining coordination structures over time.

At middle school level, several teachers highlight a lack of cohesion and coordination:

“If no one coordinates… it can’t work.” (6th grade teacher 3)

These findings indicate that, in the absence of explicit coordination mechanisms, the program struggles to achieve collective ownership within more complex organizational settings.

Teachers emphasize the need for a more formal framework to prevent the program from being perceived as peripheral or optional:

“It should be integrated into the timetable.” (6th grade teacher 1)

This reflects the necessity of formal institutionalization to ensure legitimacy and continuity, particularly in settings where activities compete with multiple curricular demands.

The issue of institutional visibility is also raised:

“To give it more importance… for example, on Pronote… so that it isn’t seen as just… the Explo’Santé recess break.” (6th grade teacher 2)

This suggests that symbolic recognition within institutional systems plays a role in shaping perceived legitimacy and engagement among both students and staff.

However, workload constraints are a potential barrier to collective membership:

“The only thing that might put colleagues off is that it’s still extremely time-consuming on our own teaching hours.” (6th grade teacher 9)

This highlights the tension between program integration and existing workload constraints, indicating that sustainability requires alignment with institutional priorities and resource allocation.

Finally, some teachers express a desire for long-term evaluative feedback:

“We’d really like to have feedback on the results. To know whether it has an impact over several years.” (6th grade teacher 11)

This points to the importance of feedback loops in sustaining engagement, suggesting that visibility of outcomes may reinforce long-term commitment among stakeholders.

Sustainability in middle school thereby depended on formal institutional embedding rather than individual initiative.

Thus, the integration of Explo’Santé appears to be shaped by structural stability, relational dynamics, organizational flexibility, and perceived symbolic legitimacy (Table 3). While primary school settings allow gradual internalization within stable classroom ecosystems, middle school environments require explicit institutional anchoring and pedagogical reframing to sustain engagement.

**Table 3:** Comparative model of program integration across primary and middle school settings.

Dimension	Primary school	Middle school
Structure	Stable	Fragmented
Organization	Individually adjustable	Collectively negotiated
Peer Climate	Gradual stabilization	Heterogeneous
Authority	Emotional proximity contained	Boundary negotiation
Engagement	Experiential, spontaneous	Conditional, relevance-based
Sustainability	Locally embedded	Requires formal institutionalization

## 4 Discussion

This study examined how Explo’Santé is perceived to shape health-supportive school environments across primary and middle school education. When considered alongside the longitudinal quantitative findings of the Explo’Santé cohort (25), the qualitative and quantitative results converge toward a coherent pattern: psychosocial gains observed during primary school appear less sustained after the transition to middle school. While differences between the 4th and the 5th grade were limited and may partly reflect normative developmental progression, the transition to the 6th grade coincided with marked discontinuities in organizational structure and relational continuity, which were associated with changes in outcome trajectories.

Globally, LS, self-efficacy, and life satisfaction increased quantitatively during primary school but declined after the transition to middle school, whereas health literacy followed a steadier upward trajectory. These differentiated trajectories raise a key theoretical question: how to conceptualize the “environment” that supports/fails to support the consolidation of LS across school stages?

Bronfenbrenner’s bioecological model (29) provides an explanatory framework to interpret these findings. Development unfolds within nested systems: the microsystem (immediate relational environment), the mesosystem (interconnections between settings), the exosystem (institutional structures), and the macrosystem (broader cultural and policy context). The qualitative findings suggest that in primary school, Explo’Santé is predominantly enacted within a stable classroom microsystem. Teachers describe continuity of adult figures, consistent spatial arrangements, and repeated symbolic routines that appear to support the gradual consolidation of emotional language and peer mediation skills. This relative stability is consistent with the upward quantitative trajectory of LS, self-efficacy, and life satisfaction during primary schooling (23, 25).

These findings align with the broader social and emotional learning literature. Meta-analytic evidence shows that universal social and emotional learning programs produce positive effects on socio-emotional skills, attitudes, and academic performance, particularly in elementary school contexts (30–32). Teacher continuity and structured classroom routines are often identified as facilitating conditions for sustained skill acquisition. Structured programs such as PATHS (33–37) or Second Step (38–41) similarly rely on repetition, modelling, and relational consistency, i.e., conditions that resemble those described by primary teachers in the present study.

However, while Bronfenbrenner’s model conceptualizes nested contextual influences, it is less operational in specifying how schools institutionalize health promotion practices. The WHO HPS framework emphasizes that sustainable health-supportive environments require integration across curriculum, leadership, governance, and community partnerships (8, 42). Thus, Bronfenbrenner offers an ecological explanation of developmental embedding (29), whereas HPS provides a systems-level implementation framework (12, 43, 44).

In primary school, the qualitative findings suggest that microsystem coherence may partially offset limited whole-school embedding. Classroom-level stability appears sufficient to sustain program routines locally, even when institutional integration is modest. The single-teacher model and contained spatial layout function as protective organizational buffers that maintain symbolic continuity despite limited formal governance integration.

In contrast, the transition to middle school highlights the potential limits of interventions that rely predominantly on microsystem-level continuity. Teachers in the 6th grade describe timetable fragmentation, spatial instability, multiple adult figures, and competing curricular demands. These characteristics correspond to exosystem-level constraints within Bronfenbrenner’s model (29) and to structural dimensions emphasized in the HPS framework (12, 43, 44). The quantitative decline in LS, self-efficacy, and life satisfaction at the end of the 6th grade (25) may therefore reflect reduced continuity of supportive relational and organizational conditions rather than a simple erosion of psychosocial competencies.

These findings raise the question of whether microsystem-level changes are sufficient to generate sustained system-level impact. While primary school settings appear to support the internalization of relational and psychosocial processes within stable classroom environments, the transition to middle school suggests that these changes may remain fragile in the absence of broader organizational and institutional alignment. This highlights the potential limits of interventions that rely predominantly on microsystem-level mechanisms, and underscores the importance of multi-level integration to achieve durable environmental transformation.

This interpretation is consistent with transition research. Longitudinal studies repeatedly show that school belonging, engagement, and life satisfaction often decline following the move to secondary education, particularly when relational continuity is disrupted (45–48). Decreases in perceived teacher warmth during this transition have been associated with reduced engagement and wellbeing (49, 50). The present qualitative findings echo these patterns: teachers describe not so much an absence of socio-emotional skills as a more fragile environment in which those skills are harder to stabilize.

Comparisons with whole-school antibullying programs further illuminate these dynamics. Interventions such as KiVa explicitly target peer norms and collective climate, recognizing that early adolescence is marked by intensified social comparison and status negotiation (51, 52). Meta-analytic evidence indicates that such programs can reduce victimization, but effects vary substantially depending on contextual and implementation factors (53, 54). The variability observed in middle school engagement in the present study is consistent with this literature: peer relevance and symbolic legitimacy appear critical during early adolescence, as illustrated by stronger engagement with topics related to social influence and by teachers’ concerns about the limited visibility of the program within institutional tools and online platforms (e.g., Pronote).

**Figure 1:**
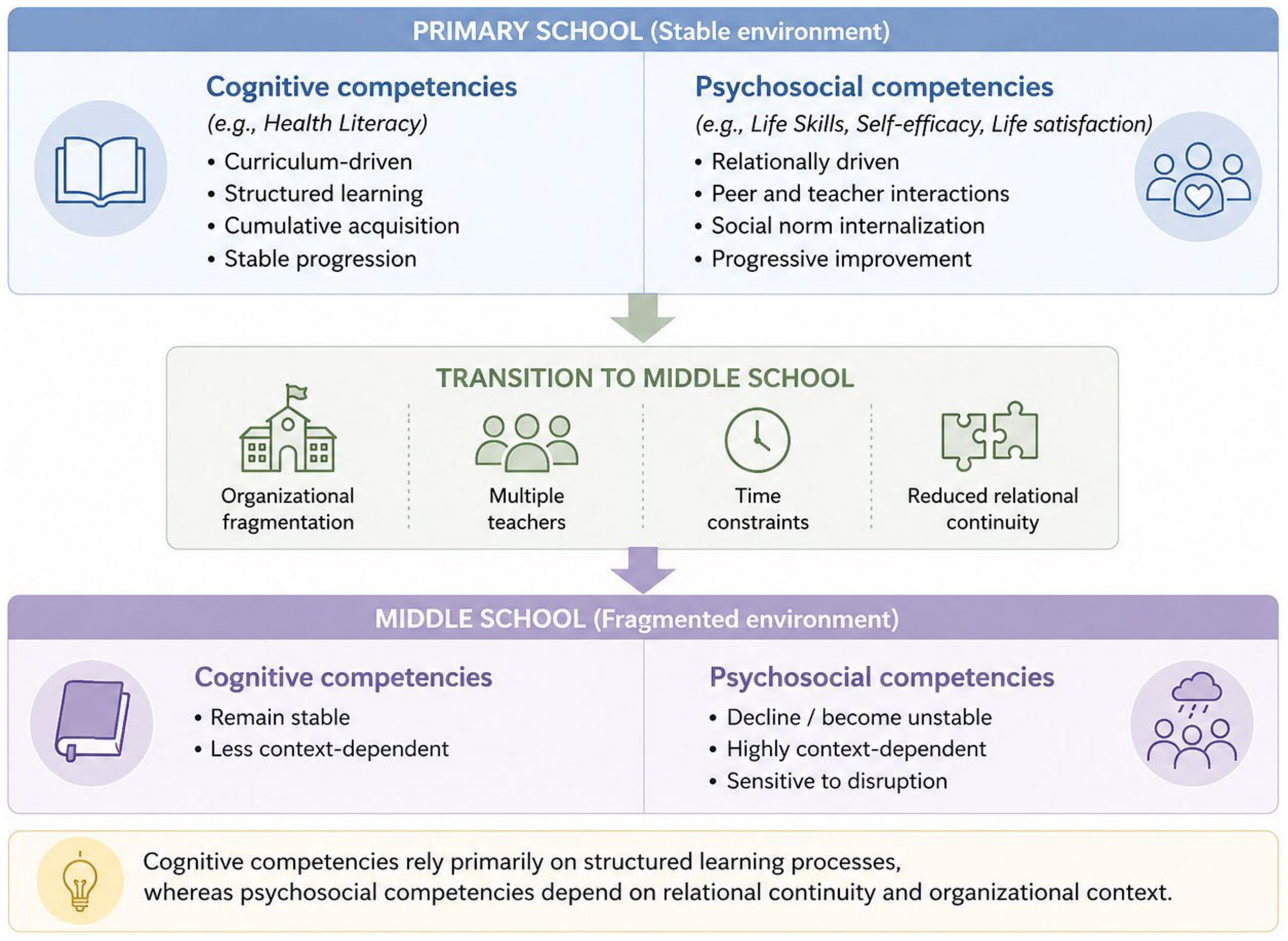
Differential pathways of cognitive and psychosocial competencies across school contexts. The figure illustrates the distinct mechanisms underlying the development of cognitive and psychosocial competencies across educational stages. Cognitive competencies (e.g., health literacy) appear primarily driven by structured and cumulative learning processes embedded within formal curricula, and remain relatively stable across the transition to middle school. In contrast, psychosocial competencies (e.g., life skills, self-efficacy, life satisfaction) rely more strongly on relational continuity and environmental conditions, making them more sensitive to organizational disruptions such as fragmentation of schedules, reduced teacher continuity, and weaker institutional integration. These differences help explain the divergence observed in outcome trajectories across school contexts.

The divergence between psychosocial indicators and health literacy further supports an ecological interpretation. Health literacy increased steadily and did not decline at the end of the 6th grade. Unlike LS or life satisfaction, health literacy may rely more heavily on cumulative instructional exposure and structured cognitive processing. Conceptual models of health literacy emphasize its progressive development across adolescence (55–57). Thus, health literacy may be relatively more curriculum-dependent, whereas LS and life satisfaction appear more sensitive to relational and organizational climate. This distinction is summarized in Figure 1, which highlights the differential pathways of cognitive and psychosocial competencies across school contexts. While cognitive competencies appear primarily supported by structured and cumulative learning processes, psychosocial competencies depend more strongly on relational continuity and organizational conditions, making them more sensitive to contextual disruptions during the transition to middle school.

The territorial disparities observed in the quantitative models resonate with findings from school health promotion research indicating that program effects are mediated by local climate, leadership, and socio-economic context (8). The qualitative data confirm that contextual conditions, e.g., training facilitation, scheduling flexibility, parental engagement, and leadership support, shape how healthy environments are enacted. Without contextual reinforcement, universal interventions risk reproducing pre-existing territorial inequalities rather than reducing them.

In this regard, realist evaluation research offers an additional interpretive lens (58). Programs function through context–mechanism–outcome configurations rather than uniform causal chains. Applied to Explo’Santé, primary school contexts appear to provide combinations of enabling conditions, such as teacher continuity, contained space, scheduling flexibility, that facilitate integration. In contrast, secondary school contexts introduce constraining combinations, with fragmented schedules, institutional compartmentalization, peer status hierarchies, that may limit dosage, continuity, and perceived relevance. Identifying recurrent “contextual equations” may therefore be more informative than assuming a standardized implementation model.

This implementation-sensitive perspective also aligns with co-creation approaches in health promotion, which stress negotiated alignment among stakeholders to sustain change (59). The present findings suggest that sustaining psychosocial gains beyond primary school may require structured coordination between primary and secondary staff, explicit transition modules, and stronger institutional recognition within secondary-school governance structures. Cross-level pedagogical articulation may represent a missing mesosystem lever in the current configuration.

Rather than interpreting the 6th grade decline as evidence of intervention failure, it may be more appropriate to view the primary-to-secondary transition as a contextual “stress test” for the continuity of health-supportive environments. Psychosocial outcomes appear environmentally contingent. Sustained effects likely depend not only on program content but on ecological fit between developmental stage, institutional framework, and organizational alignment.

This study presents several limitations. First, the longitudinal design does not involve repeated interviews with the same teachers. Consequently, findings reflect cross-cohort evolution rather than individual developmental trajectories of professional practice. This design should therefore be understood as a repeated cross-sectional qualitative approach, and findings interpreted as reflecting contextual and organizational variations across educational stages rather than individual-level change over time. Secondly, this study relies exclusively on teachers’ perspectives. Although teachers play a central role in shaping school environments, the absence of direct student voices limits the capacity to fully capture experiential and peer-level dynamics. Future research integrating students’ perspectives would provide valuable triangulation and deepen understanding of how environmental transformations are perceived by young people themselves.

## 5 Conclusion

Integrating qualitative teacher narratives with longitudinal quantitative trajectories supports an ecological interpretation of Explo’Santé’s developmental patterns. Health-supportive school environments appear to be co-constructed at the intersection of microsystem stability, institutional organization, and developmental timing. Future school health promotion strategies could therefore integrate ecological sensitivity, transition-specific reinforcement, and stronger systemic embedding to sustain psychosocial gains across educational stages.

## Data Availability

The original contributions presented in the study are included in the article/Supplementary material, further inquiries can be directed to the corresponding author/s.
