# Supplementary material for: Building health-promoting school environments: a longitudinal qualitative study of the Explo’Santé program in France
Source: Front Public Health. 2026 May 26;14:1857951. doi: 10.3389/fpubh.2026.1857951 (PMC13246645; doi:10.3389/fpubh.2026.1857951)
Supplement: Supplementary file 1 [file Supplementary_file_1.docx]

Supplementary Material

**Supplementary Table 1.** Detailed participant characteristics

| **Participant** | **Sexe** | **Grade taught** | **School context** | **Educational qualification** |
| --- | --- | --- | --- | --- |
| Teacher 1 | W | 4 | Rural | Recruitment Exam for Elementary School Teachers |
| Teacher 2 | W | 4 | Rural | Recruitment Exam for Elementary School Teachers |
| Teacher 3 | W | 4 | Rural | Recruitment Exam for Elementary School Teachers |
| Teacher 4 | W | 4 | Rural | Recruitment Exam for Elementary School Teachers |
| Teacher 5 | W | 4 | Rural | Recruitment Exam for Elementary School Teachers |
| Teacher 6 | W | 4 | Rural | Recruitment Exam for Elementary School Teachers |
| Teacher 7 | M | 4 | Rural | Recruitment Exam for Elementary School Teachers |
| Teacher 8 | M | 4 | Rural | Recruitment Exam for Elementary School Teachers |
| Teacher 9 | W | 4 | Urban | Recruitment Exam for Elementary School Teachers |
| Teacher 10 | W | 4 | Urban | Recruitment Exam for Elementary School Teachers |
| Teacher 11 | W | 4 | Rural | Recruitment Exam for Elementary School Teachers |
| Teacher 12 | W | 4 | Rural | Recruitment Exam for Elementary School Teachers |
| Teacher 13 | W | 5 | Rural | Recruitment Exam for Elementary School Teachers |
| Teacher 14 | W | 5 | Rural | Recruitment Exam for Elementary School Teachers |
| Teacher 15 | W | 5 | Rural | Recruitment Exam for Elementary School Teachers |
| Teacher 16 | W | 5 | Rural | Recruitment Exam for Elementary School Teachers |
| Teacher 17 | W | 5 | Rural | Recruitment Exam for Elementary School Teachers |
| Teacher 18 | W | 5 | Rural | Recruitment Exam for Elementary School Teachers |
| Teacher 19 | W | 5 | Rural | Recruitment Exam for Elementary School Teachers |
| Teacher 20 | M | 5 | Rural | Recruitment Exam for Elementary School Teachers |
| Teacher 21 | W | 5 | Urban | Recruitment Exam for Elementary School Teachers |
| Teacher 22 | W | 5 | Urban | Recruitment Exam for Elementary School Teachers |
| Teacher 23 | W | 5 | Rural | Recruitment Exam for Elementary School Teachers |
| Teacher 24 | W | 5 | Rural | Recruitment Exam for Elementary School Teachers |
| Teacher 25 | W | 6 | Rural | Certificate of Qualification for Secondary School Teaching |
| Teacher 26 | W | 6 | Rural | Certificate of Qualification for Secondary School Teaching |
| Teacher 27 | W | 6 | Rural | Certificate of Qualification for Secondary School Teaching |
| Teacher 28 | W | 6 | Rural | Certificate of Qualification for Secondary School Teaching |
| Teacher 29 | W | 6 | Urban | Certificate of Qualification for Secondary School Teaching |
| Teacher 30 | W | 6 | Urban | Certificate of Qualification for Secondary School Teaching |
| Teacher 31 | W | 6 | Urban | Certificate of Qualification for Secondary School Teaching |
| Teacher 32 | W | 6 | Urban | Certificate of Qualification for Secondary School Teaching |
| Teacher 33 | W | 6 | Urban | Certificate of Qualification for Secondary School Teaching |
| Teacher 34 | M | 6 | Urban | Certificate of Qualification for Secondary School Teaching |
| Teacher 35 | M | 6 | Rural | Certificate of Qualification for Secondary School Teaching |
| Teacher 36 | W | 6 | Rural | Certificate of Qualification for Secondary School Teaching |

# Supplementary Material 1. Development of the interview guide

The semi-structured interview guide was developed as part of the Explo’Santé qualitative research protocol and was designed to align with the objectives of the intervention, the theoretical frameworks underpinning the program, and the principles of implementation science (1).

Guide development was informed by the HPS framework, the Whole School Approach, and a socio-ecological perspective on health promotion, which conceptualize school health as the result of interactions between physical, social, organizational and institutional environments. In addition, the guide was structured in accordance with key dimensions of implementation research, including program adoption, implementation processes, contextual influences, perceived effects and conditions for sustainability.

Initial themes were defined deductively based on:

- the primary objective of identifying environments conducive to the implementation of Explo’Santé;
- secondary objectives related to understanding implementation processes, mechanisms of engagement, contextual facilitators and barriers, and perceived program effects beyond those captured quantitatively;
- existing literature on school-based health promotion and complex interventions.

The interview guide for teachers included open-ended questions organized around the following domains:

- introduction and contextualization of the program within the school;
- teachers’ representations and understanding of the program’s objectives;
- conditions and processes of implementation in the classroom and school setting;
- adaptations made during implementation;
- perceived effects on students, classroom climate and school environments;
- organizational, institutional and contextual facilitators and barriers;
- perceptions of engagement, sustainability and added value of the program.

Questions were intentionally phrased in a non-directive and exploratory manner, allowing participants to elaborate freely on their experiences while ensuring consistency across interviews, educational levels and data collection waves (end de 4th grade, end de 5th grade and end of 6th grade). Probing questions were used by interviewers to clarify meanings, explore examples, and deepen emerging themes when appropriate.

The interview guide was standardized across the three data collection waves, with minor adjustments in wording to account for the timing of program exposure and participants’ position in the implementation process. This approach ensured longitudinal coherence while allowing participants to reflect on both immediate and evolving experiences of the program.

The final interview guides were reviewed internally by the research team to ensure conceptual clarity, alignment with study objectives, and feasibility within the interview timeframe. All interviews were conducted using this guide as a flexible framework rather than a rigid questionnaire, consistent with qualitative research standards.

# Supplementary Material 2. Data collection

Data were collected through individual interviews conducted in France at different phases of program implementation: with 4th grade teachers between November 2023 and January 2024, with 5th grade teachers between May and July 2024, and with 6th grade teachers between April and June 2025. All interviews were conducted in a one-to-one format via videoconference using the Cisco WebEx platform by a team of three researchers (ADB, CS and MO).

At the beginning of each interview, participants were informed about the study procedures, including the objectives of the interview, data protection measures, and anonymization processes. Interviews were conducted using a semi-structured interview guide (1), which provided a common framework (2) while allowing flexibility for participants to elaborate on their experiences. Interviewers used prompts and follow-up questions to clarify responses and explore emerging topics in greater depth when appropriate.

Recordings were transcribed verbatim and subsequently deleted. Transcript accuracy was checked by an additional researcher trained in qualitative analysis. No field notes were taken during or after the interviews.

1. Olivo M, Darlington-Bernard A, Salque C, Fraticelli L, Ricard E, Carrouel F, Darlington E. The Explo’Santé mixed methods protocol: an interventional research school health promotion project in France. *Arch Public Health* (2025) 83:26. doi: 10.1186/s13690-024-01487-y

2. DeJonckheere M, Vaughn LM. Semistructured interviewing in primary care research: a balance of relationship and rigour. *Fam Med Community Health* (2019) 7:e000057. doi: 10.1136/fmch-2018-000057
